# Transcriptome-Wide Mapping of Pseudouridines: Pseudouridine Synthases Modify Specific mRNAs in *S. cerevisiae*

**DOI:** 10.1371/journal.pone.0110799

**Published:** 2014-10-29

**Authors:** Alexander F. Lovejoy, Daniel P. Riordan, Patrick O. Brown

**Affiliations:** 1 Department of Biochemistry, Stanford University School of Medicine, Stanford, California, United States of America; 2 Howard Hughes Medical Institute, Stanford University School of Medicine, Stanford, California, United States of America; 3 Department of Genetics, Stanford University School of Medicine, Stanford, California, United States of America; The John Curtin School of Medical Research, Australia

## Abstract

We developed a novel technique, called pseudouridine site identification sequencing (PSI-seq), for the transcriptome-wide mapping of pseudouridylation sites with single-base resolution from cellular RNAs based on the induced termination of reverse transcription specifically at pseudouridines following CMCT treatment. PSI-seq analysis of RNA samples from *S. cerevisiae* correctly detected all of the 43 known pseudouridines in yeast 18S and 25S ribosomal RNA with high specificity. Moreover, application of PSI-seq to the yeast transcriptome revealed the presence of site-specific pseudouridylation within dozens of mRNAs, including RPL11a, TEF1, and other genes implicated in translation. To identify the mechanisms responsible for mRNA pseudouridylation, we genetically deleted candidate pseudouridine synthase (Pus) enzymes and reconstituted their activities *in vitro*. These experiments demonstrated that the Pus1 enzyme was necessary and sufficient for pseudouridylation of RPL11a mRNA, whereas Pus4 modified TEF1 mRNA, and Pus6 pseudouridylated KAR2 mRNA. Finally, we determined that modification of RPL11a at Ψ -68 was observed in RNA from the related yeast *S. mikitae*, and Ψ -239 in TEF1 mRNA was maintained in *S. mikitae* as well as *S. pombe*, indicating that these pseudouridylations are ancient, evolutionarily conserved RNA modifications. This work establishes that site-specific pseudouridylation of eukaryotic mRNAs is a genetically programmed RNA modification that naturally occurs in multiple yeast transcripts via distinct mechanisms, suggesting that mRNA pseudouridylation may provide an important novel regulatory function. The approach and strategies that we report here should be generally applicable to the discovery of pseudouridylation, or other RNA modifications, in diverse biological contexts.

## Introduction

There is much still to be discovered about the post-transcriptional control of gene expression [1], [2]. One relatively unexplored mechanism for post-transcriptional regulation of gene expression is chemical modification of mRNAs [3], [4]. Covalent modification of RNAs has been studied for decades and dozens of chemically-distinct RNA modifications, affecting hundreds of different nucleotides in non-coding RNAs have been found; many of these modifications are conserved throughout all kingdoms of life [5].

While nucleotide modifications in non-coding RNAs are well known, relatively little is known about possible modifications in mRNAs, and even less is known about what roles these modifications could play. The only established nucleotide modifications in mRNAs, outside of the 5′-cap complex, are N6-methyladenosine [6] and 5-methylcytosine [7]. While N6-methyladenosine has been known to occur in mRNAs for nearly 40 years [8], only in the past few years have locations of N6-methyladenosines in mammalian mRNAs been mapped [9], [10]. Methylation of the N6 position of specific adenosines in mRNA is essential for sporulation in *S. cerevisiae* [11], [12]. The role this modification plays in gene expression is still a subject of debate. Multiple studies have shown N6-methyladenosine has an effect on binding of RNA binding proteins [9], [13], [14], but there are conflicting results on the effect of N6-methyladenosine on mRNA translation [15], [16] and on stability of modified transcripts [4], [14], [17].

Three transcriptome-wide screens for a second covalent modification of mRNA bases - cytosine 5-methylation – have identified about 10,000 such sites in human mRNAs [7], [18], [19]. The function of 5-methylcytosines in mRNA is still unknown.

The ability to identify modification of mRNAs at specific sites opens a search for a potential new molecular mechanism by which gene expression could be regulated. Are there other modified based in mRNAs, in addition to N6-methyladenosine and 5-methylcytosine? The single most abundant modified RNA base is pseudouridine (Ψ), a structural isomer of uridine that has been found in tRNAs, rRNA, and snRNA [20]. In *S. cerevisiae*, many pseudouridines are present in tRNAs, nearly 50 pseudouridines have been found in rRNA, and 8 have been identified in snRNAs [3]. While pseudouridylation is known to have functions in ribosome biogenesis [21], [22], protein synthesis [23], translation accuracy [24], and pre-mRNA splicing [25], the specific roles of most individual pseudouridines are unknown. Pseudouridine does not differ from uridine in its base-pairing specificity, but the subtle difference between these two nucleotides can affect the stability of base pairs, base-stacking interactions and RNA tertiary structure [26], [27]. Pseudouridine can be distinguished from uridine in RNA by virtue of its specific chemical properties. The most commonly used way to identify pseudouridines in non-coding RNAs relies on its ability to be selectively modified by 1-cyclohexyl-(2-morpholinoethyl)carbodiimide metho-p-toluene sulfonate (CMCT). When RNA is treated with CMCT, the carbodiimide (CMC) moiety modifies the N1 of guanosine, the N3 of uridine, and the N1 and N3 of pseudouridine [28]. An alkaline treatment at pH 10.3 removes CMC from all these sites except for the N3 of pseudouridine. The bulky CMC modification acts as a barrier to reverse transcription through the modified base [29]. Treatment of RNA with CMCT followed by reverse transcription with gene-specific primers has thus been used to identify and map sites of pseudouridylation in non-coding RNAs [30], [31].

Most of the known RNA modifications are constitutive, but a few pseudouridines can be induced in snRNAs in yeast under certain stress conditions–specifically, in heat shock and in stationary phase [32]. These induced pseudouridines have been proposed to alter regulation of splicing patterns under these stress conditions. Although pseudouridines have not been reported to occur naturally in mRNA, a recent experiment using an artificial system in yeast showed that pseudouridylation of a stop codon can increase the rate of translational readthrough [33], suggesting that pseudouridylation might influence gene expression post-transcriptionally.

In this study, we developed pseudouridine site identification sequencing (PSI-seq), a high-throughput sequencing technique based on the CMCT reverse-transcription stop assay to identify pseudouridines in mRNAs, which we used to systematically characterize the pseudouridine content of the yeast transcriptome.

## Results

### High-Throughput Sequencing Method for Transcriptome-wide Identification of Pseudouridines

**Figure 1:**
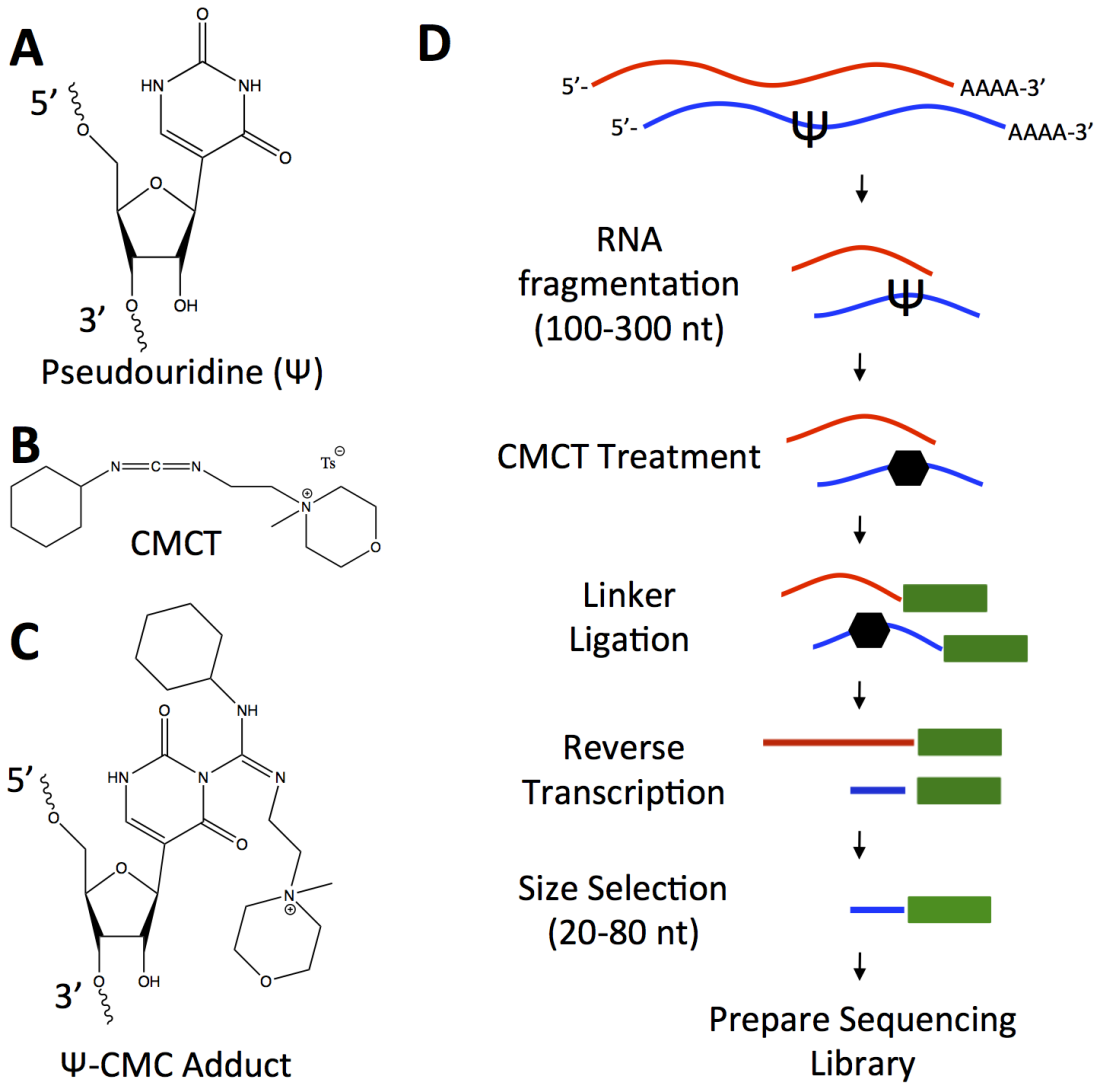
Overview of Pseudouridine Site Identification Sequencing (PSI-Seq). Chemical structures of pseudouridine (A) and CMCT (1-cyclohexyl-(2-morpholinoethyl)carbodiimide metho-p-toluene sulfonate) (B) are shown, as well as the CMC-adduct (C) generated by reaction of pseudouridine with CMCT followed by alkaline treatment. The bulky CMC group can stop reverse transcription of RNA. (D) Diagram of library preparation strategy for sequencing assay to identify pseudouridines in cellular RNA. The RNA sample is fragmented by alkaline hydrolysis and size-selected for RNA pieces 100 to 300 nt in length. RNA fragments are then reacted with CMCT to produce CMC-adducts (shown as a black hexagon). In parallel, the same sample is incubated with buffer lacking CMCT (mock treatment) as a negative control. RNA is then alkaline treated to remove adducts from non-pseudouridines. Linkers are ligated onto the 3′-end of the RNA fragments to allow for reverse transcription. Reverse transcription from the linkers is performed and cDNA is isolated. Size selection for truncated cDNA products with insert lengths of 20-80 nt is performed, yielding only cDNAs whose reverse transcription stopped before the end of the RNA fragment. These cDNAs are converted into sequencing libraries using a protocol adapted from the ribosome profiling technique (Ingolia et al. 2010).

Our strategy for identifying for pseudouridylated RNAs was based on the ability of chemical treatment with CMCT to modify pseudouridines specifically (Figure 1a–c), and the ability of the resulting carbodiimide-modified bases to stop reverse transcription at sites of modification. We first fragmented oligo(dT)-purified poly-adenylated yeast RNA by base hydrolysis, then purified fragments 100 to 300 nucleotides in length. We treated the resulting RNA fragments with CMCT, followed by alkaline treatment to remove CMC from uridines and guanosines, leaving the CMC modification only on pseudouridines. We then ligated a specific adapter oligonucleotide onto the 3′-end of the RNA fragments, to serve as a priming site for reverse transcription. After reverse transcription of these fragments, we selected for cDNAs in which reverse transcription stopped before the end of the RNA fragment by selecting for primer extension products corresponding to inserts less than 90 nt in length (i.e. much smaller than the template RNA fragments from which they were derived) (Figure 1d). We then used a protocol similar to that used for ribosome profiling to prepare these libraries for high-throughput sequencing [34]. Because the library preparation protocol positions a sequencing primer immediately adjacent to the 3′ end of the cDNA, the first base of the sequencing reads should correspond to the last base incorporated by reverse transcription. If the 3′-end of the cDNA results from a stop in reverse transcription at a carbodiimide-modified pseudouridine, the first base of our sequencing read should delineate the base immediately 3′ to a pseudouridine in the mRNA sequence. Thus, this method should, in principle, allow us to map pseudouridines with single-nucleotide precision.

We isolated RNA from yeast grown in rich media to log phase, and from this RNA, we prepared and sequenced four libraries. The first was made using the pseudouridine-specific CMCT/alkali protocol as described above using natural RNA isolated from yeast; three control libraries were as follows: 1. A library produced by the identical procedure, with the exception that CMCT treatment was omitted (mock control), 2. A library prepared by the procedure described above, starting with RNA transcribed *in vitro* from amplified copies of all annotated yeast transcripts, 3. A library prepared identically to library 2, but omitting CMCT treatment. For bona fide sites of pseudouridylation, we would expect to see a surplus of sequencing reads representing cDNA products with 5′-ends one base upstream of a pseudouridine in the corresponding yeast RNA molecule, exclusively in the library made from CMCT-treated natural yeast RNA. Because some pseudouridines in noncoding RNAs are specifically induced by heat shock or in stationary phase, we analyzed two samples of RNA isolated from log phase yeast and one each from heat shocked yeast and yeast in stationary phase, and corresponding controls.

### Method Validation: Pseudouridines in Ribosomal RNA

**Figure 2:**
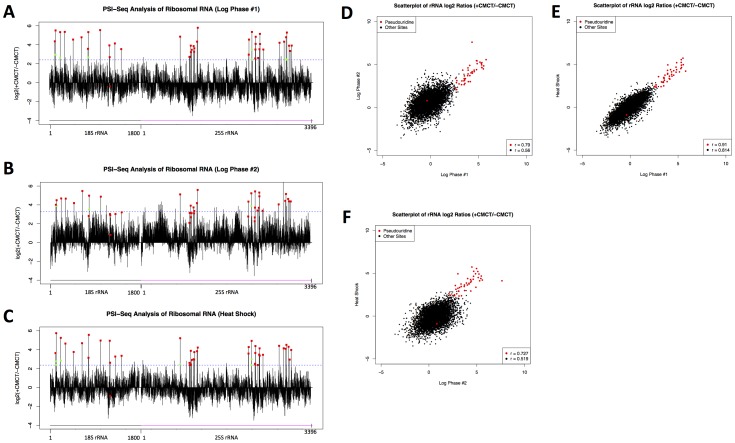
PSI-Seq Identifies Known Pseudouridine Sites in Yeast Ribosomal RNA. For each nucleotide in 18S and 25S rRNA, the log2 ratio of the number of reads (plus one) stopping at that position in the +CMCT library versus the –CMCT (mock) library was plotted. The dashed blue line indicates the cutoff for the top 60 sites in each condition, which includes all 43 known pseudouridine sites (red squares) that are not further modified. Sites among the top 60 that were located exactly one away from known pseudouridines, representing “secondary stops”, are marked with green circles. The data are plotted for yeast RNA isolated from both log phase (A–B) and heat shock (C) conditions. Scatterplots of the log_2_ ratios shown in (A–C) are displayed for pairwise comparisons to assess reproducibility of values for known pseudouridines (red) and all sites (black) between independent experiments (D–F).

A small percentage of residual ribosomal RNA remaining in our RNA samples, even after two rounds of poly(A) selection, enabled us to use the well-mapped pseudouridines in rRNA as a set of internal positive controls. For each nucleotide in the 18S or 25S ribosomal RNA (5196 nucleotides total), we calculated the ratio of reads ending one nucleotide upstream in the library from CMCT-treated yeast RNA, to corresponding reads in the library from the same yeast RNA without CMCT treatment. We would expect sites of pseudouridylation to have the highest values for this ratio, and this is what we found –for log-phase replicate #1 and for the heat shock condition, all 43 of the known pseudouridine sites in rRNA were among the top 60 nucleotides with the highest values for this ratio, whereas 30/43 pseudouridines were included in the top 60 sites for log-phase replicate #2 (38/43 in the top 120 sites) and 25/43 for the stationary phase sample (30/43 in top 120) (Figure 2, Table S1, Figure S1 in File S2). These data show that PSI-seq is the first method capable of identifying at single-nucleotide resolution all the pseudouridines in rRNA in a single experiment. Among the bases with the 60 highest +/− CMCT ratios in each sample, but not previously known pseudouridines, several (20 sites) are bases adjacent to known pseudouridines– an expected result, as secondary stops one nucleotide away have been seen in previous reverse-transcription stop experiments using CMCT to identify pseudouridines [35]. Notably, the secondary stops adjacent to known pseudouridines that we detected occurred at positions both one base 5′ and one base 3′ of the modifications, indicating that CMC-induced polymerase stalling can specifically occur before and after (as well as at) sites of modification. Overall comparison of the log2 ratios (+CMCT/-CMCT) for known pseudouridine sites across pairs of conditions showed that they were highly correlated (with a mean Pearson's correlation coefficient value of 0.79, and all pairwise *p*-values <10^−15^) and were better correlated than the values from non-pseudouridines, demonstrating significant reproducibility of the results between independent samples (Figure 2). In the libraries from the stationary cells, known pseudouridine sites were less-well enriched, with the 43 sites included only among the top 792 nucleotides with the highest +/− CMCT abundance ratios (Figure S2 in File S2). Due to this lower technical quality, we exclusively focused on the data from log phase cells (replicates 1 & 2) and heat shocked cells for the rest of the study.

### Determination of Candidate Pseudouridines in mRNAs

**Figure 3:**
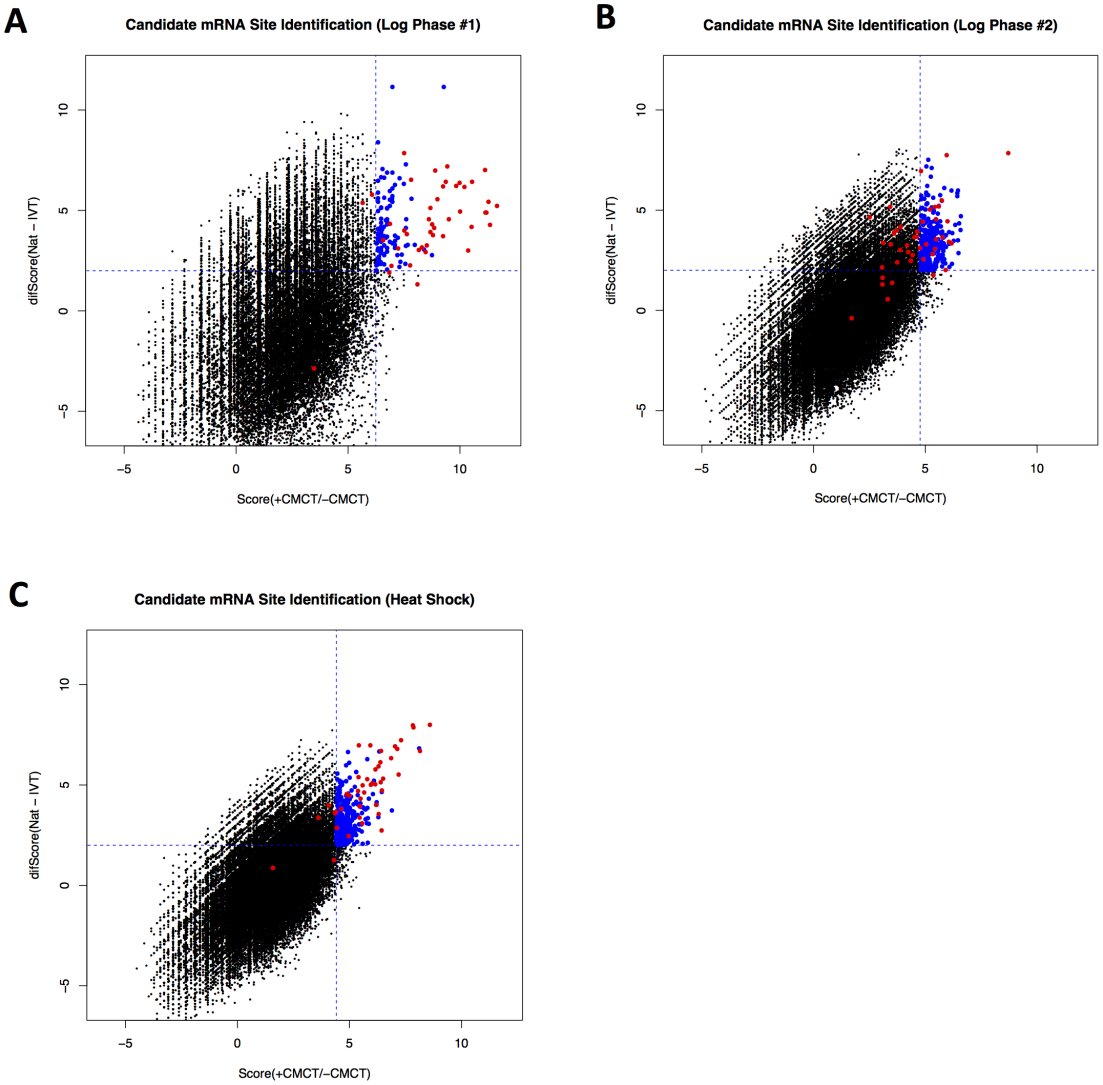
PSI-Seq Analysis of the Yeast Transcriptome Reveals mRNA Pseudouridylation Sites. (A–B). For RNA isolated from yeast growing in log phase (A–B) or heat shock (C) conditions, pseudouridylation “scores” were calculated based on a regression analysis of the log2 transformed normalized read densities for each nucleotide position in treated (+CMCT) versus mock (-CMCT) samples (details in text). Values plotted on the x-axis indicate scores (+CMCT/−CMCT) from PSI-seq analysis of natural RNA samples. Values plotted on the y-axis indicate the difference between the natural RNA (+CMCT/−CMCT) scores and the corresponding score values obtained from PSI-seq analysis of unmodified *in vitro* transcribed control RNA samples. Each data point corresponds to a single nucleotide position within the transcriptome. Vertical and horizontal dashed blue lines illustrate the cutoff thresholds used as criteria for candidate pseudouridine identification for each experiment. Unique non-rRNA-derived uridine sites in the upper-right quadrant were classified as candidate mRNA pseudouridines (blue circles). Known pseudouridine sites from the ribosomal RNA are also shown (red circles).

We next considered the possibility that site-specific pseudouridines may occur in mRNAs. In a genome-wide comparison of relative frequencies of individual nucleotides immediately downstream of sites where reverse transcription of yeast RNAs stopped, we observed outliers represented by many more reads in the libraries from CMCT-treated RNA than in the –CMCT library (Figure 2). Most of these outliers corresponded to sites of known pseudouridines in rRNA (represented by red squares), but a few corresponded to nucleotides in mRNAs. Two prominent examples (marked with arrows in Figure 3) were nt 68 in the RPL11a coding sequence, and nt 239 in the TEF1/TEF2 coding sequence (note that the sequence flanking nt 239 is identical between TEF1 and TEF2; thus we cannot tell whether the modification occurred in TEF1 mRNAs, TEF2 mRNAs, or both).

To systematically identify candidate pseudouridylation sites, we calculated a best-fit linear model for the log_2_-transformed number of reads terminating at a specific nucleotide in the CMCT-treated sample, as a function of the log_2_-transformed number of reads terminating at that nucleotide in the mock-treated RNA sample. For each nucleotide, we calculated the difference between the observed and model-predicted values for the +CMCT condition, and divided this quantity by the standard deviation of the observed vs. predicted residuals. We then ranked the nucleotides based on this “score”. We considered a nucleotide to be a candidate pseudouridylation site if its score exceeded a threshold defined as the highest score observed for any rRNA nucleotide that was not identical or adjacent to a known pseudouridylation site, corresponding to a false positive rate of about 0.0002. In the data from log phase yeast, 41/43 (replicate 1) and 20/43 (replicate 2) of the known rRNA pseudouridines scored above this threshold, and in the data from heat-shocked yeast, 38/43 rRNA pseudouridines exceeded this threshold. To further filter the candidate pseudouridylation sites, we excluded nucleotides with scores that were not at least four-fold greater (i.e. a difference in log_2_ scores of more than 2.0) than the corresponding “score” obtained for the *in vitro* transcribed RNA control data (where the block to reverse transcription cannot be ascribed to a pseudouridine), and positions derived from non-uridine bases were also eliminated (Figure 3). After these additional criteria were applied and redundant or ribosomal RNA-derived sites were removed, there remained 103 unique candidate pseudouridine sites in 56 different mRNAs from the log-phase replicate #1 data, 335 sites (150 mRNAs) from the log-phase replicate #2 data, and 335 candidate sites (208 mRNAs) from the heat-shock data. Candidate sites of pseudouridylation are listed in table S2. The number of candidate sites exactly identified as pseudouridines in multiple experimental conditions was modest, likely owing to the technical challenges of globally detecting pseudouridylation events with high sensitivity from lowly expressed transcripts. Nevertheless, the observed degree of overlap was extremely significant (5 sites in both log-phase #1 and #2, *p*  =  1e-7; 9 sites in both log-phase #1 and heat-shock, *p*  =  3e-15; 14 sites in both log-phase #2 and heat-shock, *p*  =  2e-18; all *p*-values computed by hypergeometric distribution), due to the vast number of possible nucleotides evaluated from throughout the transcriptome (Figure 3, Table 1). Moreover, candidate sites identified in one condition showed significantly elevated values for their log_2_(+CMCT/−CMCT) scores in other conditions, suggesting that differences in identification of these sites are at least partly due to the precise classification thresholds we adopted (Table S1). Overall, these results suggest the existence of naturally occurring pseudouridylation at precise internal sites within potentially hundreds of specific mRNAs in yeast (Table S1). Notably, two sites were consistently identified as high-confidence pseudouridine candidates in all three of the examined samples: the 68^th^ nucleotide in the RPL11a coding sequence (RPL11a nt 68), and the 239^th^ nt in the coding sequence of TEF1/2. Because the two strongest candidate pseudouridine sites derived from two genes with clear functional roles in translation, we performed gene ontology (GO) analysis to search for functional themes that were enriched among the genes we identified as harboring pseudouridylation sites. Interestingly, significant GO term enrichment for the category “cytoplasmic translation” was observed for the set of genes with candidate sites from log-phase growth (75 out of 180 genes, *p*  =  5.93e-76) as well as heat-shock (71 out of 208 genes, p  =  8.03e-64), suggesting that pseudouridylation may provide a mechanism for coordinately regulating these functionally related mRNAs. However, it is also worth noting that PSI-seq analysis is inherently biased towards detecting sites in more abundant transcripts (due to sampling effects), which may be a confounding factor for GO analysis.

**Table 1:** Candidate mRNA Pseudouridine Sites Identified in Multiple Samples.

Site	Log #1	Log #2	HS	Gene	nt	Region	Name
chr16:731679	***9.278***	***6.465***	***8.116***	YPR102C	67	CDS	RPL11A
chr16:700830	***7.601***	***4.857***	***6.445***	YPR080W	238	CDS	TEF1
chr15:980500	5.811	***5.550***	***6.893***	YORCd25	92	N/A	YORCΔ25
chr13:24202	***7.835***	4.427	***5.301***	YML123C	1599	CDS	PHO84
chr1:72955	***7.342***	***5.389***	4.377	YAL038W	1168	CDS	CDC19
chr7:735635	5.811	***5.550***	***5.346***	YGR122C-A	128	CDS	YGR122C-A
chr15:60739	4.688	***6.197***	***5.796***	YOL139C	285	CDS	CDC33
chr7:482839	***7.584***	3.101	***5.562***	YGL008C	−168	5-UTR	PMA1
chr10:383159	***8.756***	2.305	***4.992***	YJL034W	1837	CDS	KAR2
chr2:300266	***6.986***	3.837	***4.877***	YBR031W	100	CDS	RPL4A
chr12:370000	***7.226***	***5.492***	2.443	YLR110C	99	CDS	CCW12
chr13:632425	4.491	***5.422***	***5.212***	YMR186W	71	CDS	HSC82
chr15:159531	5.171	***4.988***	***4.751***	YOL086C	1063	3-UTR	ADH1
chr13:220170	***6.339***	***5.089***	3.352	YML029W	2808	3-UTR	USA1
chr4:356857	4.688	***4.867***	***5.118***	YDL055C	−98	5-UTR	PSA1
chr16:642498	4.004	***5.354***	***5.305***	YPR035W	293	CDS	GLN1
chr11:327012	***6.309***	3.534	***4.502***	YKL060C	119	CDS	FBA1
chr13:24315	***7.097***	1.877	***4.761***	YML123C	1486	CDS	PHO84
chr1:73018	***6.348***	2.473	***4.773***	YAL038W	1231	CDS	CDC19
chr16:702042	4.004	***4.914***	***4.619***	YPR080W	1450	3-UTR	TEF1
chr11:164375	2.988	***5.089***	***5.165***	YKL152C	11	CDS	GPM1
chr4:556458	3.170	***5.004***	***4.742***	YDR050C	12	CDS	TPI1
chr13:511403	3.335	***5.047***	***4.463***	YMR122W-A	89	CDS	YMR122W-A
chr2:89115	−0.262	***5.487***	***5.869***	YBL072C	8	CDS	RPS8A

List of the 24 candidate mRNA pseudouridine sites from ψ-seq analysis of yeast RNA that were independently identified in multiple experiments from log phase and heat shock RNA samples. The table lists the yeast systematic name (Gene) and standard name (Name), whether the site occurs in the coding domain sequence (CDS) or 5′ (5-UTR) or 3′ untranslated regions (3-UTR), and the position of the site relative to the start of the ORF (nt). The pseudouridylation “score” computed for each site is shown for each of the three experiments analyzed (Log #1  =  log-phase replicate #1, Log #2  =  log-phase replicate #2, HS  =  heat shock), with ***bold and italicized*** scores depicting the sample from which each site was identified as a candidate mRNA pseudouridine.

There was no discernable bias in the location of putative pseudouridines relative to canonical features of mRNAs (Figure S3 in File S2) nor, for pseudouridines within coding sequences, was there a bias in the position of the pseudouridine within the affected codon.

### Identification of Enzymes Responsible for Site-Specific mRNA Modification

Initially, we considered the possibility that pseudouridylation of mRNA might be directed by H/ACA snoRNAs, as it is in rRNA. For each of the putative pseudouridines we identified, we examined the sequence surrounding the candidate modified site for complementarity against all known guide regions in yeast snoRNAs, looking for 8–17 nt of base-pairing compatibility between the guide region and mRNA target, allowing up to three mismatches (since pseudouridylation of U2 snRNAs can tolerate up to three mismatches with the cognate snoRNA [32]). Based on this analysis, none of the candidate mRNA pseudouridines appeared to be obviously targeted for modification by any known snoRNAs.

We searched the putative pseudouridylation sites for sequences that might direct modification by known tRNA pseudouridine synthases. We focused initially on the two strongest candidates from our sequencing data: nt 68 in RPL11a and nt 239 in TEF1/2. A six nucleotide sequence containing nt 68 in RPL11a - UCUGUU (where the underlined U is the potential pseudouridine) - matched a six nucleotide sequence containing a known pseudouridine–nt 44 in U2 snRNA, whose synthesis is catalyzed by the pseudouridine synthase Pus1p [36], making Pus1p a leading candidate for catalyzing the modification in RPL11a. In TEF1/2, the sequence GUUCGA, which contains nt 239 (underlined), matches a sequence in the TΨC loop in tRNAs that includes the eponymous, highly conserved pseudouridine. The TΨC loop pseudouridylation is catalyzed by Pus4p [37], so we hypothesized that Pus4p might also modify nt239 of TEF1/2.

**Figure 4:**
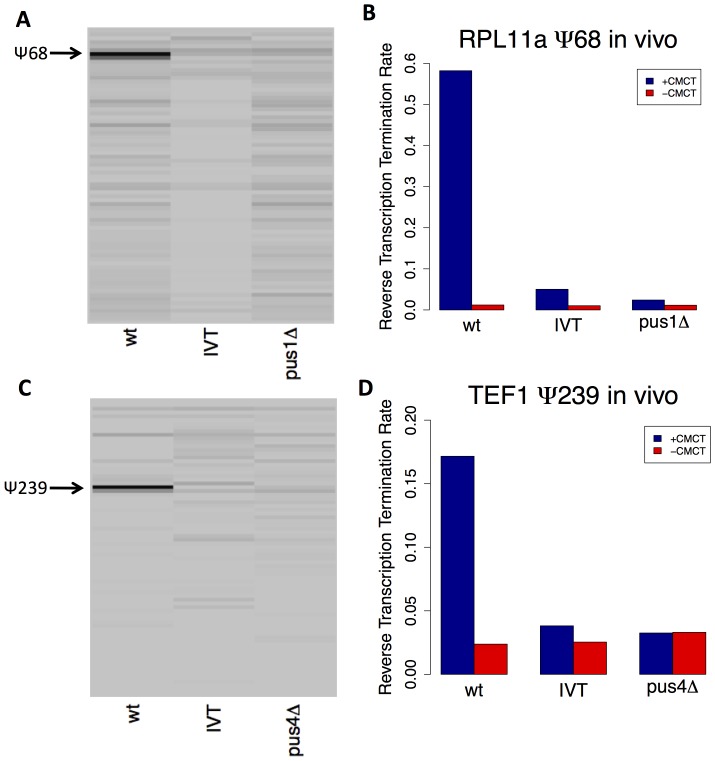
Primer Extension Analysis of Pseudouridine Sites in RPL11a and TEF1 mRNAs. Reverse transcription using fluorescent gene-specific primers for RPL11a (A–B) or TEF1 (C–D) was performed on RNA samples following treatment with CMCT (+CMCT) or mock reactions (-CMCT), and analyzed by capillary electrophoresis. At each position, the difference in primer termination rates between +CMCT and –CMCT conditions was computed from electropherogram intensities and is displayed in a gel-like format. Positions appearing with darker intensities correspond to sites with specific termination induced by CMCT treatment, as expected for pseudouridines. RNA samples examined were natural RNA isolated from wild-type yeast (wt), *in vitro* transcribed RNA as an unmodified control (IVT), or natural RNA isolated from a mutant yeast strain (pus1Δ or pus4Δ). The site of pseudouridylation is marked by an arrow. Quantification of the site-specific termination rate for each RNA sample is plotted for RPL11a psi-68 (B) and TEF1 psi-239 (D).

To test these hypotheses, we analyzed RNA from wild type (BY4741), pus1Δ or pus4Δ yeast with a CMCT RT-stop assay using fluorescently-labeled gene-specific primers, and evaluating the primer extension products by capillary electrophoresis. The corresponding RNA sequences, transcribed *in vitro* with unmodified NTPs, served as negative control templates, while synthetic RNAs containing pseudouridine at the predicted, *in vivo* sites served as positive controls. We observed the predicted RT stops in both RPL11a and TEF1 RNAs isolated from wild type yeast (Figure 4). With a substrate RNA isolated from the pus1Δ strain, the RT stop was seen at the predicted site in TEF1, but absent from RPL11a, indicating that detectable pseudouridylation of base 68 in RPL11a mRNA requires Pus1p. Similarly, in the products of reverse transcription of RNA from the pus4Δ strain, the RT stop was seen absent at the predicted site in TEF1, demonstrating that Pus4p is necessary for the pseudouridylation of site 239 in TEF1 mRNA. These results suggest that Pus1 and Pus4 directly modify RPL11a and TEF1 mRNAs, respectively, and further validate the identity of the modified sites as pseudouridine.

Based on standard curves constructed by applying the RT-stop assay to mixtures of the negative and positive controls at known ratios (Figure S4 in File S2), we crudely estimated that in rapidly dividing wild-type yeast, about 40% of RPL11a transcripts and about 60% of TEF1/2 transcripts contained a pseudouridine at the predicted sites. We also purified RPL11a and TEF1 mRNA from total yeast RNA, digested these mRNAs to individual nucleotides, and performed mass spectroscopy to independently corroborate the presence of pseudouridine in the mRNA species that were modified. Both RPL11a and TEF1 mRNA samples show pseudouridine levels above background, consistent with the mRNA species being pseudouridylated (described in Supplemental text in File S1 and Figure S5 in File S2).

### Specific Pseudouridylation of RPL11a and TEF1 mRNAs Using Purified Pseudouridine Synthases

**Figure 5:**
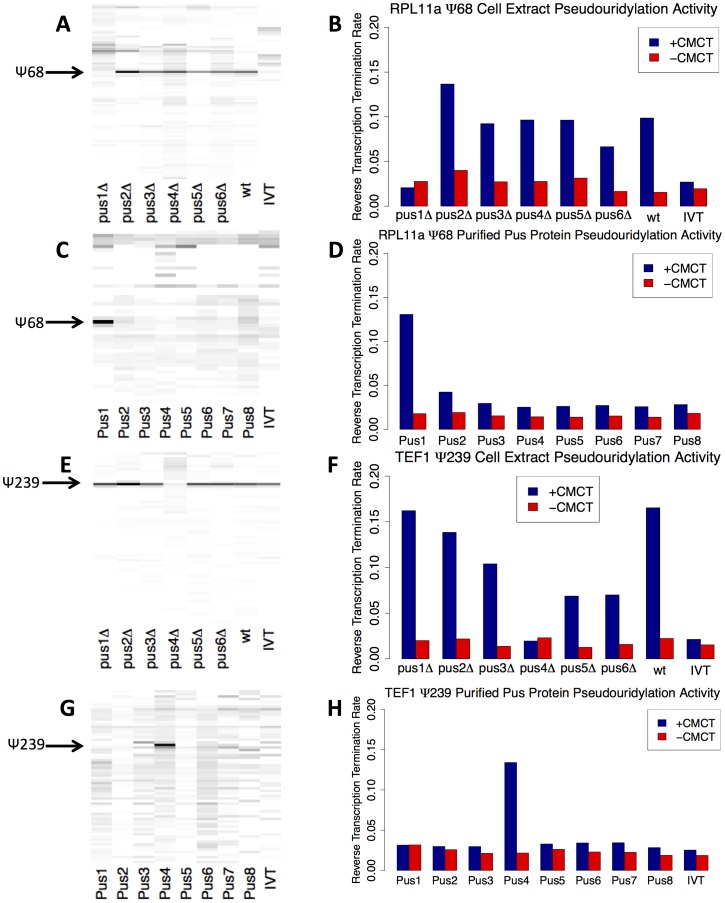
RPL11a RNA and TEF1 RNA Can Be Pseudouridylated by Cell Extract and Purified Pseudouridine Synthases. A. Unmodified *in vitro* transcribed RPL11a RNA was either left untreated (IVT) or was incubated with cell extract made from wild-type yeast (wt) or mutant yeast lacking an individual pseudouridine synthase gene (pus1-pus6). The RNA was then analyzed by reverse transcription primer extension with or without CMCT treatment, and the data were plotted as described in figure 4. B. For these modification experiments, the proportion of reverse transcription that stopped at the site of pseudouridylation was plotted for both +CMCT (blue) and mock (red) conditions as described in figure 4B. C. *In vitro* transcribed RPL11a RNA was modified by purified pseudouridine synthase enzymes Pus1p through Pus8p, or untreated (IVT). The CMCT-RT stop experiment was performed, and the data were plotted as described in figure 4A. D. For these experiments, the proportion of reverse transcription that stopped at the site of pseudouridylation was plotted for both +CMCT (blue) and mock (red) conditions as described in figure 4B. E-H. Same as A-D, for TEF1 in vitro transcribed RNA.

To directly verify the identity of the specific pseudouridine synthases responsible for the observed RNA modifications, we developed an *in vitro* pseudouridylation activity assay based on incubation of unmodified RPL11a and TEF1 mRNAs, produced by *in vitro* transcription, with yeast cell extracts. Using the RT-stop-capillary electropheresis assay, we found that both RPL11a and TEF1 were specifically modified at the sites of *in vivo* pseudouridylation in this *in vitro* system (Figure 5a,b,e,f). To test which pseudouridine synthases were necessary for modification of the two transcripts, the same *in vitro* modification assays were carried out using extracts from yeast strains with each of 7 pseudouridine synthases, respectively, knocked out. As expected from our *in vivo* analysis of natural yeast mRNA, Pus4p was specifically required for modification of TEF1 mRNA (Figure 5e,f), and Pus1p was specifically required for modification of RPL11a mRNA (Figure 5a,b).

We also purified eight hexahistidine-tagged pseudouridine synthases (Pus1p-Pus8p) by nickel affinity chromatography from yeast strains in which each was overexpressed. The partially purified proteins (Figure S6 in File S2) were each assayed for their ability to modify IVT RPL11a mRNA or TEF1 mRNA *in vitro*. Only Pus4p was able to modify TEF1 mRNA, and only Pus1p was able to modify RPL11a mRNA (Figure 5g,h,c,d).

We used this same *in vitro* modification system to evaluate additional mRNAs suspected to be pseudouridylated based on our genome-wide screen. We obtained data for three additional mRNAs, YTM1 site 185, KAR2 site 1916, and CDC33 site 286, synthesized by *in vitro* transcription, for specific pseudouridylation *in vitro* by each of the 8 purified Pus proteins. For each of these three transcripts, we confirmed specific pseudouridylation by one pseudouridine synthase: YTM1 mRNA was modified by Pus1p, KAR2 mRNA was modified by Pus4p, and CDC33 mRNA was modified by Pus6p (Figure S7 in File S2). In these cases, the pseudouridine synthases that performed modification matched those suggested by sequence context of the putative pseudouridylation site. The sequence around the putative pseudouridine at position 185 in YTM1 mRNA is CUGUU, which matches the sequence modified by Pus1p in U2 snRNA; the sequence around the putative pseudouridine at position 1916 in KAR2 mRNA, GUUCGA, matches the motif modified by Pus4p. In addition, the predicted secondary structure around the putative pseudouridine at position 286 in CDC33 mRNA matches the secondary structure surrounding sites in tRNAs modified by Pus6p. Since we were able to identify the enzymes that modified these sites based on their sequence context, we used the known motifs of some pseudouridine synthases to search our sequencing data (defined by these motifs) specifically for CMCT-dependent RT stops at the candidate sites, to try to identify more mRNA pseudouridines (see File S1). We found a partial but significant enrichment of stops at these sites, indicating that these motifs are likely involved in directing pseudouridylation, but other features likely contribute as well.

### Sequence Elements Involved in mRNA Pseudouridylation

**Figure 6:**
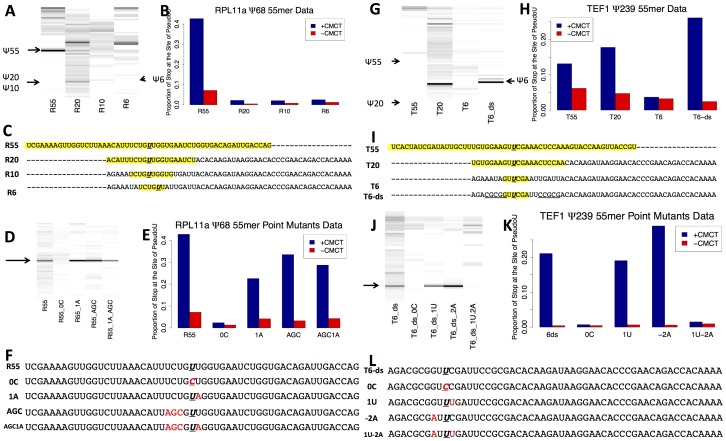
Determination of Sequence Requirements for Pseudouridylation of RPL11a and TEF1 mRNAs. A. 55 nt RNA constructs containing 55 nt (R55), 20 nt (R20), 10 nt (R10), or 6 nt (R6) of sequence corresponding to the sequence around the site of modification in RPL11a mRNA were *in vitro* transcribed and modified with purified Pus1p. The RNA was then used for the CMCT-RT stop experiment, and the data were plotted as described in figure 3A. B. For these modification experiments, the proportion of reverse transcription that stopped at the site of pseudouridylation was plotted for both +CMCT (blue) and mock (red) conditions as described in figure 3B. C. The sequences of the constructs used. For R20, R10, and R6, the sequences conserved from RPL11a are in blue. D. 55 nt constructs of containing the 55 nt of sequence surrounding the site of modification in RPL11a mRNA were *in vitro* transcribed with the following mutants: no mutation (R55), site of pseudouridylation (U68) to C (R55_0C), U69A (R55_1A), UCU(65-67) to AGC (R55_AGC) or both U69A and UCU->AGC (R55_AGC1A). The RNA was then used for the CMCT-RT stop experiment, and the data were plotted as described in figure 3A. E. For these modification experiments, the proportion of reverse transcription that stopped at the site of pseudouridylation was plotted for both +CMCT (blue) and mock (red) conditions as described in figure 3B. F. The sequences of the constructs used. The mutated nucleotides are in red. G-L. Same as A-F, for the following TEF1 constructs: 55 nt around the site of pseudouridylation in TEF1 (T55), 20 nt around the site (T20), 6 nt around the site (T6), 6 nt around the site in a 5 nt stem loop (T6-ds), T6ds with the U that is modified mutated to C (T6_ds_0C), the C 1 nt downstream mutated to U (T6_ds_1U), the G 2 nt upstream mutated to A (T6_ds_-2A) or both those mutations (T6_ds_1U-2A). For the sequence of T6-ds, the complementary nucleotides comprising the 5 nt stem are underlined.

To better define the sequences required to specify pseudouridylation of mRNAs we synthesized 75 nt RNA oligonucleotides and assayed them for *in vitro* pseudouridylation by Pus1p or Pus4p, using the RT-stop, capillary electrophoresis assay. The 3′ 20 nucleotides of each of these test substrates were complementary to a fluorescently labeled oligonucleotide that served as a primer for reverse transcription. The remaining 55 nt of each of the synthetic substrates contained portions of the sequence surrounding the pseudouridylation sites in RPL11a or TEF1, with arbitrary “filler” sequences making up the remainder. For RPL11a, the sequences we tested contained 55 nt (R55), 20 nt (R20), 10 nt (R10) surrounding the *in vivo* modification site, or just the 6 nt motif that matches the site of modification in U2 snRNA (R6). For TEF1, the sequences we tested contained 55 nt (T55) or 20 nt (T20) from the sequence surrounding the *in vivo* TEF1 pseudouridine, or just the 6 nt motif that matches the site of modification in tRNAs (T6). Previous work showed that modification by Pus4 requires a stem-loop structure in its target RNA [33], so we tested a fourth model substrate in which the 6 nt motif containing the site of modification was positioned in a loop with a 5 nt double-stranded stem with a different sequence but similar predicted structure to the TEF1 modification site (T6-ds). We tested each of these model substrates for pseudouridylation by purified Pus1p (for RPL11a model substrates) or Pus4p (for TEF1 model substrates), and used the RT stop assay to detect and quantitate the specific modification (Figure 6 a,b,e,f). For both transcripts, the model substrates containing 55 nt from the native site were efficiently modified. The model RPL11a substrates with less than 55 nt from the RPL11a transcript were not efficiently modified. The 20 nt TEF1 model substrate containing only 20 nt from the native site, and the T6-ds model substrate were modified as efficiently as the 55 nt construct, while the TEF1 model containing the 6 nt sequence without a flanking stem remained unmodified, suggesting that the 6 nt motif is sufficient for modification in the context of the correct secondary structure, but that the secondary structure is also required. It was also clear from the high-throughput sequencing data that the 6 nt motif alone was not enough for modification; it occurs frequently in the transcriptome, but there was no evidence for pseudouridylation at most of these sites. In fact, there was no evidence for modification of a second GUUCGA motif, which occurs without any apparent a stem-loop structure, in the TEF1 transcript itself.

To identify specific nucleotides important for directing pseudouridylation, we mutated nucleotides surrounding the site of modification in the R55 and T6-ds model substrates. In the R55 substrate, we replaced the sequence UCUGUU with UCUGCU (R55-0C, lacking the modified uridine - a negative control), UCUGUA (R55-1A), AGCGUU (R55-AGC), and AGCGUA (R55-AGC1A). In the T6-ds substrate, we replaced the sequence GUUCGA with GUCCGA (T6-ds-0C, lacking the modified uridine - a negative control), GUUUGA (T6-ds-1U), AUUCGA (T6-ds- -2A), and AUUUGA (T6-ds-1U-2A). Apart from the negative controls, each of the tested mutations was synonymous. The negative controls, as expected, showed no evidence of pseudouridylation in our *in vitro* assay (Figure 6 c,d,g,h). All the other mutant substrates were modified to an extent comparable to the positive controls (R55 and T6-ds) except for T6-ds-1U-2A, in which no modification was detected.

The *in vitro* data suggest that GUUCGA and CUGUU contribute to the cis elements that direct pseudouridylation of TEF1/2 and RPL11a mRNAs, respectively, but that they are not enough to specify sites for pseudouridylation. We analyzed our genome-wide RT-stop sequencing data to see if they support this idea. Using more inclusive guidelines to define a site as a CMC-dependent stop (> 10 reads in natural +CMCT and –CMCT conditions combined, +CMCT natural/−CMCT natural > 3, and +CMCT natural/−CMCT natural > 3 * +CMCT IVT/−CMCT IVT), we compared the rate of CMC-dependent stop at every 7-mer with U in the 4^th^ position. We could use these data to look at enrichment of CMC-dependent stops in specific 5- and 6-mers as well. Compared to all possible 6-mers with U in the 3^rd^ position, GUUCGA sites are somewhat enriched for CMCT dependent stops in the heat shock data (4/463, p  =  0.010). Compared to all possible 5-mers with U in the 4^th^ position, CUGUU are also somewhat enriched for CMCT dependent stops in the heat shock data (30/6376, p  =  0.0028). However, neither of these oligomers were enriched for CMCT-dependent stops in the log phase data. These data support the idea that these sequences alone do not direct modification of specific sites in the transcriptome, but they may play a part in directing pseudouridylation.

### Evolutionary Conservation of mRNA Pseudouridylation

Having identified sites of modification in Saccharomyces mRNAs, we investigated whether the mRNA pseudouridines we identified in *S. cerevisiae* were conserved in other species. First, we aligned the sequences for RPL11a mRNA and TEF1 with their available mRNA orthologues in eight fungal species (*Saccharomyces cerevisiae*, *Saccharomyces paradoxus*, *Saccharomyces mikitae*, *Saccharomyces bayanus*, *Saccharomyces castellii*, *Candida albicans*, *Neurospora crassa*, and *Schizosaccharomyces pombe*) (Figure S8 in File S2). In both cases, the site of pseudouridylation in *S. cerevisiae* remained a uridine in every species. In the 6-nucleotide sequence around the site of modification in RPL11a mRNA that matches the six nucleotides around the site of modification by Pus1p in U2 snRNA (UCUGUU), the first U is conserved in all seven species, the nucleotides in positions 2-6 are conserved in six species, and the final U is conserved in four species. In TEF1's orthologues, the entire six-nucleotide motif (GUUCGA) is conserved in all seven species, except in *C. albicans*, where the first G is replaced with an A.

**Figure 7:**
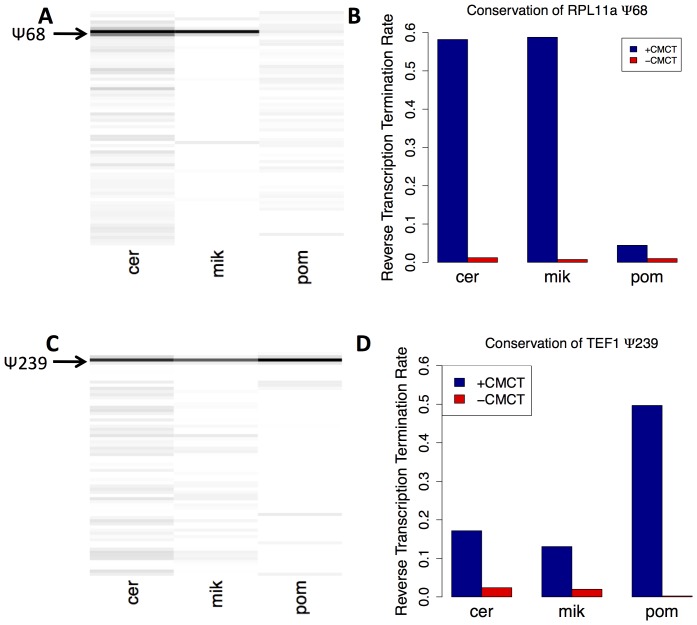
Pseudouridylation of RPL11a and TEF1 mRNAs is Conserved in Other Fungal Species. A,B. The CMCT-Stop experiment was performed on the orthologues of RPL11a in *Saccharomyces mikitae* and *Schizosaccharomyces pombe*. The proportion of reverse transcription that stopped at the site of pseudouridylation was plotted for both +CMCT (blue) and mock (red) conditions as described in figure 3A and B. C,D. As in A,B, for the orthologues of TEF1 in *S. mikitae* and in *S. pombe*.

To determine whether the pseudouridine itself was conserved at the orthologous sites in the other fungi, we performed the RT-stop capillary sequencing assay on the RPL11a and TEF1 orthologues in mRNA from *S. mikitae*, *S. pombe*, and *S. cerevisiae* (Figure 7). For RPL11a, the modification was conserved in *S. mikitae*, but not in *S. pombe*. For TEF1, the modification was conserved in both *S. mikitae* and *S. pombe*. The last common ancestor of *S. cerevisiae* and *S. pombe* is estimated to have lived 600 million years ago [38], suggesting that this pseudouridine in TEF1 mRNA is an ancient mRNA modification that may confer an evolutionary fitness advantage.

### Functional consequences of mRNA Pseudouridylation

In an artificial system in yeast, pseudouridylation of stop codons has been found to cause increased read-through of stop codons [33], suggesting that pseudouridylation of mRNA could change a transcript's coding potential. Using a mass-spectroscopy-based approach, we were unable to detect a change in the amino acid encoded by the modified codons in RPL11a or TEF1. (See File S1 for details). To investigate whether pseudouridylation of these mRNAs affected the abundance of the RNA or the encoded protein, we took advantage of the pus1Δ and pus4Δ yeast strains, in which RPL11a and TEF1 mRNAs, respectively, are unmodified. However, we were unable to find any significant changes in RPL11a or TEF1 mRNA or protein abundance in these strains relative to wild-type (See File S1 and Figure S9 in File S2 for details).

## Discussion

Covalent modifications of non-coding RNAs have been recognized for more than 50 years, but have rarely been investigated at the genome-wide level [39]. Apart from the 5′ cap structure, the only site-specific covalent modifications of mRNA identified to date are N6-methyladenosine and 5-methylcytosine. Because the classical methods used to search for such modifications have limited sensitivity, we suspected that additional modes of modification could have escaped detection. To explore this possibility, we developed a deep sequencing-based technique to identify sites of pseudouridylation in mRNAs. Application of this method to mRNA from yeast uncovered direct evidence for naturally occurring site-specific pseudouridylation of potentially hundreds of mRNAs *in vivo*, with mRNAs encoding genes involved in translation showing preferential enrichment. Because the sensitivity of PSI-seq analysis is dependent on sequencing depth, it seems likely that additional pseudouridylation events occurring in rare transcripts (or specific physiological conditions) may have escaped detection. The pseudouridines we identified may therefore be only the “tip of the iceberg” in terms of the broader landscape of transcriptome-wide chemical diversity of RNA.

Because of the versatility of the snoRNA programmed H/ACA snoRNP system, we initially considered it a prime suspect for the mRNA modifications. All of the modifications we characterized in detail, however, were catalyzed by pseudouridine synthases that were previously thought to modify only tRNAs and snRNAs at specific sites. We found three such enzymes that introduce pseudouridines into mRNA in yeast, based on the loss of specific pseudouridine modifications *in vivo* in knockout mutant strains lacking Pus1, Pus4 and Pus6, respectively. We confirmed the roles of these three enzymes biochemically by recapitulating the reactions *in vitro*, using partially purified enzymes and synthetic substrates.

While numerous pseudouridines and other covalent modifications of noncoding RNAs have been appreciated for many years, their specific functions are, in most cases, still undefined. We eagerly sought to uncover the functional roles of the pseudouridine sites we identified in yeast mRNAs, but a clear role for these modifications in regulating gene expression has remained elusive. Given the strong functional coherence of the pseudouridylated mRNAs involved in translation and protein biosynthesis, as well as the extensive evolutionary conservation of the modifications to RPL11a and TEF1, we suspect that the pseudouridylation events we have uncovered are likely adaptive. Although we were unable to detect any clear effects of these pseudouridylation events on mRNA stability, translation rate, or protein coding potential, we nevertheless anticipate that future investigations into the functional consequences of these RNA modifications will shed light on this intriguing possibility. Considering the growing awareness of the previously unappreciated chemical diversity of cellular mRNAs, it is tempting to speculate that extensive combinations of covalent mRNA modifications may represent an additional layer of post-transcriptional regulatory information modulating the transcriptome, analogous to the way that DNA and histone modifications epigenetically control genome function.

The PSI-seq method we developed to search for pseudouridines in RNA should be broadly applicable to finding such sites in other species, and for comprehensive identification of the RNA targets of specific pseudouridine synthases. Although we have only so far discovered mRNA pseudouridines in fungi, there is no reason to believe they are unique to these species. Indeed, the presence of pseudouridine modifications in the regulatory non-coding RNA, SRA, in humans [40], and the unexpected developmental phenotype of mutations in a pseudouridine synthase in Toxoplasma gondii [41], suggest that a systematic global search for pseudouridines in these and other species may lead to important discoveries.

## Methods

### Strains and Growth Conditions

Unless otherwise stated, the strain used in this study was BY4741 grown in YPD at 30°C to an OD_600_ of 0.6-0.8 for “log phase” yeast. For heat shock experiments, cells were grown to an OD_600_ of 0.6-0.8, harvested by centrifugation at 3000 x g for 5 min, resuspended in YPD media preheated to 45°C, and then incubated at 45°C for an additional 30 minutes. For stationary phase experiments, cells were grown to an OD_600_ of 0.6-0.8 at 30°C, then grown for another ∼24 hours to an OD_600_ of ∼4-6. TAP-tagged yeast strains were derived from BY4741 (Open Biosystems Cat# YSC1177). Purification of Pus proteins was done using moveable ORF strains (Open Biosystems). Knockout strains were a gift from the Davis lab. Pus knockout, TAP-tag strains were made by genomically inserting TAP tags into the Pus knockout strains, then selecting on his- plates, according to standard yeast genetics techniques.

### Oligonucleotides

Any oligonucleotides used in this study are in File S3.

### Isolation of RNA for High-Throughput Sequencing

2x1L of yeast were grown to log phase. Half of each liter was harvested immediately, while the other 500 mL of one L was heat shocked, and 500 mL of the other liter was taken to stationary phase. The cells were harvested by centrifugation, and the pellet was washed in 5 mL ice-cold TES buffer (10 mM Tris-HCl pH 7.5, 1 mM EDTA, 0.5% SDS), then resuspended in 1–2 mL TES buffer. An equal volume of acid phenol-chloroform was added to each resuspended pellet, then the mixture was heated to 65°C for one hour with vortexing every 10 minutes. The mixtures were spun for 5 min at 4°C, then the aqueous phase was isolated. An equal volume of chloroform was added, the mixtures were vortexed and spun down for 5 min at 4°C. The aqueous phase was isolated, 1/10 volume of 3 M NaOAc and an equal volume of isopropanol were added, and the RNA was precipitated and resuspended in 1–2 mL water. For each condition, 300 µg of RNA was subjected to two rounds of poly(A) purification using the Poly(A) Purist MAG kit (Ambion), giving about 3–6 µg of poly(A) RNA. The poly(A) purified RNA was isopropanol precipitated and resuspended in 20 µL water.

To generate transcriptome-wide samples of unmodified *in vitro* transcribed (IVT) RNA, the following procedure was performed. 1 µg of total RNA was used as input for the MessageAmp II aRNA amplification kit (Ambion) to make antisense RNA (aRNA). A short poly(A) tail was added to the aRNA using ePAP (Invitrogen), then that RNA was used as input for the MessageAmp II aRNA kit to give unmodified sense RNA. 18S and 25S rDNA were amplified from genomic DNA using primers that added at T7 promoter at the 5′ end of the DNA. Unmodified 18S and 25S rRNA were transcribed from these constructs using the MEGAScript *in vitro* transcription kit (Ambion). Unmodified IVT 18S and 25S rRNA were spiked into the IVT samples.

### Library Preparation for High Throughput Sequencing to Identify Pseudouridylation in mRNA

To 3 µg (20 µL) of the poly(A) purified RNA, 20 µL of 2x alkaline hydrolysis buffer (10 mM Na2CO3, 90 mM NaHCO3, pH≈9.3) was added, and the RNA was fragmented for 6.5 minutes at 98°C, then isopropanol precipitated and resuspended in 40 µL water. The sample was then split in half–one +CMCT sample and one mock sample. To each sample was added 100 µL BEU buffer (7 M urea, 4 mM EDTA, 50 mM bicine pH 8.5) with (+CMCT) or without (mock) 200 mM 1-cyclohexyl-(2-morpholinoethyl)carbodiimide metho-p-toluene sulfonate (CMCT, Sigma). These samples were incubated at 37°C for 20 minutes, then precipitated and resuspended in sodium carbonate buffer (50 mM sodium carbonate, 2 mM EDTA) and incubated at 37°C for 4 hours. The RNA was then isopropanol precipitated and washed 2x in 70% EtOH, then resuspended in 5 µL water. 5 µL 2x denaturing loading buffer [34] was added, the samples were heated to 98°C for 5 minutes, then run on a 5% TBE-Urea gel for 1 hr at 150 V. The gel was stained with SYBR gold (Invitrogen), and bands containing RNA of length 100 to 300 nt were excised from the gel. The gel slice was physically disrupted and incubated at 4°C overnight, rotating in 500 µL 0.3 M NaCl. The remaining gel debris was removed using Spin-X columns (Corning), and eluted RNA samples were isopropanol precipitated and resuspended in 43 µL water.

The library preparation protocol is adapted from the ribosome footprinting library preparation [34], with changes in the sizes of RNA, cDNA, and DNA extracted from gels. Exact conditions are described in the Supplementary Methods (File S1). Samples were quantified by Agilent Bioanalyzer, and each of the four samples (+CMCT and mock, natural and IVT) for one growth condition were combined at equal concentrations, then run on the Illumina GA II for high-throughput sequencing.

### Analysis of Sequencing Data

Data were aligned to the yeast genome using Bowtie, with no more than 2 mismatches per read, and with reads that mapped to more than one genomic locus randomly assigned to one locus with equal probability. Ribosomal RNA reads at the same loci were combined, and moved to separate rRNA reads files. The number of reads mapping to each site in the genome were counted. For each condition, a scatterplot of ln(number of reads + 1) in the mock condition vs. +CMC condition was made using R. Pseudouridines in rRNA were marked with red squares. For each of these scatterplots, a regression line was calculated. For each site, the y-distance from the regression line (residual) was calculated, and it was divided by the standard deviation of all residuals to get what we referred to as a score. In order to define potential sites of pseudouridylation (hits), we used the site in rRNA with the highest score (other than sites of pseudouridylation or sites one nucleotide downstream of these sites) to determine a cutoff–anything with scores greater than these sites was considered a hit. The cutoffs used were 6.26 for log phase replicate 1, 5.52 for log phase replicate 2, 4.47 for heat shock, and 7.38 for stationary phase. To limit our lists of potential pseudouridines to sites more likely to be pseudouridines, any nts that were not Us were discarded, and any sites with scores for the IVT data within 2 of the sites score for the natural data were discarded. This left 103 sites for log phase replicate 1, 141 sites for replicate 2, 273 sites for heat shock, and 42 sties for stationary phase. Raw files and processed data have been uploaded to GEO with the accession number GSE60445.

### Purification of Overexpressed Pseudouridine Synthases

Moveable ORF strains (Open Biosystems) were used to overexpress pseudouridine synthase enzymes (Pus1p-Pus8p). Strains were grown at 30°C to OD_600_ 1.2 in 200 mL SC-Ura with 2% raffinose, then 100 mL 3x YP-6% galactose was added, and cells were shaken for 6 hours at 30°C. Cells were pelleted, washed in water, and resuspended in 2 mL lysis buffer (50 mM Hepes pH 8.0, 300 mM NaCl, 10 mM imidazole). Cell extract was made by bead beating 4x 1 min, then spinning down the lysate to remove debris. Proteins were purified from these extracts using Qiagen Ni-NTA protein purification kits, then resuspended in elution buffer (50 mM Hepes pH 8.0, 300 mM NaCl, 250 mM imidazole) plus 10% glycerol at 0.5–1 mg/mL total protein. To check purification, samples were run on SDS-PAGE gels stained with GelCode blue (Pierce).

### *In vitro* Pseudouridylation of *in vitro* Transcribed RNA

TEF1 and RPL11a genes were amplified from genomic DNA using PCR with primers that added a T7 binding site at the start of the 5′-UTR and 20 As at the end of the 3′-UTR. MEGAScript (Ambion) was used according to manufacturers' instructions to make *in vitro* transcribed RNA. 2–10 pmol of IVT RNA was added to yeast extract (0.01–1 mg/mL) or purified pseudouridine synthase enzymes (0.05–0.1 mg/mL) in 100 mM Tris-HCl pH 8.0, 100 mM ammonium acetate, 5 mM MgCl_2_, 2 mM DTT, 0.1 mM EDTA and incubated for 1 hr at 30°C. RNA was isolated by acid phenol-chloroform extraction and isopropanol precipitation, then resuspended in water.

### CMC Treatment, RT Stop-Capillary Sequencing

RT-stop capillary sequencing protocols were adapted from the Das lab Mutate-and-Map protocols [42]. As input for the reverse transcription stop-capillary sequencing experiments, 1–10 pmol RNA in 4–20 µL of water was used. To each sample, 5x volume of BEU buffer with or without 200 mM CMCT was added and incubated at 37°C for 20 minutes. The samples were isopropanol precipitated, resuspended in 50 µL 50 mM sodium carbonate buffer, then incubated at 37°C for 4 hours. These samples were isopropanol precipitated and resuspended in 2 µL water. To each sample were added 1 µL 375 mM KCl, 1 µL 1 mM EDTA, 0.75 µL water, and 0.25 µL 0.25 µM fluorescently-labeled primer. This was heated to 90°C for 5 minutes, then equilibrated at 50°C for 2–10 minutes. To these samples were added 2 µL 5x FS buffer (Invitrogen), 0.5 µL 0.1 M DTT, 0.5 µL 10 mM dNTPs, 1 µL 375 mM KCl, 0.8 µL water or 2 mM ddNTPs (for sequencing ladders), and 0.2 µL Superscript III (Invitrogen). These samples were heated at 42°C for 30 minutes, 4 µL 1 M NaOH was added and they were heated to 90°C for 5 minutes, then put on ice for 5 minutes. The samples were neutralized using 6 µL acid quench buffer (1.43 M NaCl, 1.29 M NaOAc, 0.57 M HCl), then 1.5 µL Oligo(dT) MAG beads (Ambion) were added. Samples were incubated for 10 minutes, then put on magnets and washed 2x with 40 µL 70% EtOH. Samples were dried, then resuspended in 11 µL ROX sample buffer (6 µL ROX 350 in 1.1 mL Hi-Di Formamide (ABI)) and put on a magnet. The liquid was removed to an optical plate and run on an ABI3100 capillary sequencer.

### Analysis of Capillary Sequencing Data

RT-stop capillary sequencing data were processed using the HiTrace package for Matlab [43]. Quicklook was used to view the relative intensities of stops (like a gel with radiolabeled primers). HiTrace was also used to get background-subtracted quantification of peaks corresponding to each nucleotide surrounding sites of modification. These values for the site of modification were plotted in barplots using the R script plot_proportions.r. In addition, the mock values were subtracted from the +CMC data, then plotted in R using the script plot_graph.r.

### Isolation of mRNA from different Yeast Species

4 L each of *S. cerevisiae*, *S. mikitae*, *C. albicans*, and *S. pombe* were grown to an OD_600_ of 0.8–1.0 in YPD (or YES for *S. pombe*) at 30°C. The yeast was then pelleted, washed in water, and each pellet was resuspended in 40 mL of TES buffer. For east yeast species, RNA was extracted by hot acid-phenol chloroform extraction (4x10 mL acid phenol-chloroform), and re-extracted with 10 mL chloroform. The RNA was ethanol precipitated and washed with 70% EtOH, and resuspended in 25–30 mL. For each species, total RNA was purified once over a 0.5 g Oligo(dT) celluose column, using the conditions explained above. This poly(A) RNA was then ethanol precipitated and the pellet was washed with 70% EtOH, dried, and resuspended in 100 uL water. These samples were then used in the CMCT-RT-CapSeq assay described above.

## Supporting Information

Table S1 PSI-seq read counts data for ribosomal RNA.
(XLSX)

Table S2 PSI-seq read counts data for mRNA candidate sites.
(XLSX)

File S1 Supplemental text and methods.
(DOCX)

File S2 Supporting figures S1-S9.
(TIFF)

File S3 List and description of oligonucleotides used in this study.
(XLSX)
