# Supplementary material for: Transcriptome-Wide Mapping of Pseudouridines: Pseudouridine Synthases Modify Specific mRNAs in S. cerevisiae
Source: PLoS One. 2014 Oct 29;9(10):e110799. doi: 10.1371/journal.pone.0110799 (PMC4212993; doi:10.1371/journal.pone.0110799)
Supplement: File S1 — Supplemental text and methods. (DOCX) [file pone.0110799.s003.docx]

**Supplemental Text**

**Mass Spectroscopy to Validate RPL11a and TEF1 Transcript Modification**

To get an independent validation of pseudouridine in each mRNA, we used mass spectrometry. After following the same enrichment strategy to get purified natural mRNAs, the quantities of each mRNA, as well as 18S rRNA and 25S rRNA were determined by RT-qPCR. “Background” and “positive control” samples were made—the background samples contained total yeast RNA with the same amount of ribosomal RNA as were present in the mRNA samples (determined by qPCR), and the positive control samples were the background samples plus synthetic RNA oligos with one known pseudouridine. The RNA samples digested with P1 nuclease and phosphatase treated, then LC-MS/MS was performed to quantify how much pseudouridine was present in each samples, compared to the quantities of each 2’-O-methyl nucleotide, which act as a proxy for the background (Figure S5 in File S1). The data collected are consistent with pseudouridine being present in the mRNA transcripts, with less than one pseudouridine present per transcript on average.

**Looking for mRNA Pseudouridylations at Known Pseudouridine Synthase Motifs**

If we narrow our search for sites of pseudouridylation to only those sites surrounded by known motifs for pseudouridine synthases, we can increase our ability to detect pseudouridines. We used the following four motifs to narrow our search (pseudouridines underlined): CUGUU (Pus1p), GUUCGA (Pus4p), GUUGG (Pus5p), and UNUAR (Pus7p). To identify CMCT-dependent stops at these sites, we used a more inclusive cutoff than for our previous analyses. For a given condition, any site with more than 10 reads combined between the natural +CMCT and -CMCT conditions, a natural +CMCT/natural –CMCT ratio of at least 3, and a (natural +CMCT/natural –CMCT) / (IVT +CMCT/IVT –CMCT) ratio of at least 3 was called a potential pseudouridine. We first tried to determine if any of these motifs were significantly enriched for CMCT-dependent stops in either the log phase or the heat shock condition, compared to all motifs of the same length with a U at the expected position. Of the 8 condition-motif pairs we tested, only the Pus1p motif (30/6376, p = 0.0028) and the Pus4p motif (4/463, p = 0.010) were enriched with a p-value < 0.05.

While the motifs were not generally enriched for CMCT-dependent stops, we wanted to try to find specific sites that provide evidence for modification, so we made lists of all the sites that fit the conditions listed above. These lists are provided in the appendix, and there are some interesting features of the lists. A number of the top hits from the initial analysis of the sequencing results appear to be modified by Pus1p. Along with RPL11a and YTM1, RPL4a, RPS8a, EMP24, GLN1, and GDH1 are likely modified by Pus1. Pus7 has a number of potential targets, with the strongest evidence for its modification of SPI1 mRNA. There were very few sites that appeared to be modified by Pus5p, but there is some evidence suggesting it may modify RPS15 and CIS3 mRNAs. Additionally, in these analyses, a few sites in Ty elements arose. In particular, Pus7p may modify sites in Ty elements, though this could just be an artifact of the analysis of the high-throughput sequencing results. Further studies will need to be performed to determine if any of the sites identified by these analyses represent actual pseudouridylation.

**Changes in the Amino Acids Encoded by the Modified Codons Are Not Detected by Mass Spectroscopy**

To test whether the modifications in RPL11a mRNA or TEF1 mRNA might also change the amino acid encoded by the affected codons, we cultured yeast with genetically tagged protein-A fusions of RPL11a and TEF1, respectively, and harvested in log phase, after a heat shock, and in stationary phase. We used the protein-A tag to purify the tagged proteins, further resolved them by SDS-polyacrylamide gel electrophoresis,, excised the bands corresponding to the respective tagged proteins and analyzed their amino-acid sequences by protease digestion followed by mass spectroscopy. We specifically searched for any evidence of a change (relative to the standard genetic code) in the amino acid encoded by the codon containing pseudouridine. In RPL11a, a valine at residue 23 is predicted to be encoded by the unmodified codon GUU, where the second U is modified to pseudouridine, and in TEF1, a phenylalanine at position 68 is predicted to be encoded by the unmodified codon UUC at the modification site, where the second U is modified to pseudouridine. We used an error-tolerant analysis to identify any fragments that might contain either an amino acid different from that predicted from the genomic sequence or one with a post-translational modification. For each protein, in each condition, more than 50 identified fragments matched the predicted amino acid sequence, but we found no change in the amino acid encoded by the modified codon in any of these fragments.

**No Significant Changes in RPL11a or TEF1 RNA or Protein Abundance Are Found in pus1Δ or pus4Δ Strains**

To investigate whether pseudouridylation of these mRNAs affected the abundance of the RNA or the encoded protein, we took advantage of the pus1Δ and pus4Δ yeast strains, in which RPL11a and TEF1 mRNAs, respectively, are unmodified. We isolated RNA from three replicates each of wt, pus1Δ, and pus4Δ strains grown to log phase, then performed RT-qPCR to measure the abundance of RPL11a and TEF1, and normalized their abundances to the level of ACT1 (as an internal reference standard). Neither RPL11a mRNA or TEF1 mRNA changed in abundance in the relevant knockout strains as compared to the corresponding wild-type (Figure S9a in File S1), suggesting that pseudouridylation does not effect the abundance of either of these mRNAs in exponentially growing cells.

To determine whether pseudouridylation of RPL11a or TEF1 mRNA affected the expression levels of the Rpl11a or Tef1 proteins, we introduced a gene encoding TAP-tagged RPL11a into isogenic Pus1+ and pus1Δ strains, and a gene encoding TAP-tagged TEF1 into isogenic Pus4+ and pus4Δ strains. Each resulting strain was grown in duplicate to log phase, or to log phase followed by heat shock, or to stationary phase. From each strain, under each condition, we separated proteins by SDS-PAGE, then quantitated the TAP-tagged RPL11a or TEF1 proteins by quantitative western blotting with an antibody specific to the TAP tag. Act1p or Tub1p were used as loading controls for TEF1 and RPL11a, respectively. We found no significant difference in abundance of either protein between the wild-type strain and the cognate pseudouridine synthase knockout strain, in any of the culture conditions (Figure S9b-e in File S1).

**Supplemental Methods**

**High-Throughput Sequencing Library Preparation Adapted from Ribosome Footprinting**

The samples were heated at 70°C for 90 seconds, then equilibrated at 37°C. To each sample, 5 μL 10x PNK buffer (NEB), 1 μL Superasin (Ambion), and 1 μL T4 PNK (NEB) were added, and the sample was heated at 37°C for 1 hr, then the PNK was inactivated at 75°C for 10 minutes. The sample was isopropanol precipitated and resuspended in 5 μL 10 mM Tris pH 8, then transferred to a new tube with 2 μL water and 3 μL (750 ng) Linker-1 (NEB). This was heated to 80°C for 2 minutes, then cooled to room temperature. The linker was ligated onto the 3’ end of the RNA fragment using RNA ligase 2 (NEB) in 10 μL sample volumes according to manufacturers instructions, then isopropanol precipitated and resuspended in 5 μL water. 5 uL 2x denaturing loading buffer was added, the sample was heated to 98°C for 2 minutes, then run on a 5% TBE-urea gel at 150 V for 1 hr. The gel was SYBR gold stained, then RNA of length 120 to 320 nt was extracted, and the slice was physically disrupted and incubated at 4°C overnight, rotating in 500 μL 0.3M NaCl. The liquid was isolated using Spin-X columns, then isopropanol precipitated and resuspended in 6.75 μL primer binding mix (2 μL 1.25 μM NI-NI-9 primer, 1 μL 10 mM dNTPs, 3.75 μL water). This was transferred to PCR tubes and incubated at 90°C for 5 minutes, then 3.25 μL reverse transcription premix was added (2 μL 5x cDNA synthesis buffer (Invitrogen), 0.5 μL 0.1 M DTT, 0.5 μL Thermoscript (Invitrogen), 0.25 μL Superasin (Invitrogen)) and the reverse transcription was performed at 42°C for 1 hr. To remove the RNA, 1.1 μL of 1M NaOH was added and the samples were incubated at 98°C for 20 minutes. The samples were isopropanol precipitated, then resuspended in 5 μL 10 mM Tris-HCl pH 8. 5 μL of 2x denaturing loading buffer was added, and the samples were heated at 98°C for 2 minutes and run on a 5% TBE-urea gel at 150 V for ~1 hr. The gel was SYBR gold stained, then cDNA of length 130 to 180 nt (cDNA where the reverse transcription stopped before the end of the RNA fragment—insert sizes from ~30-80 nt) was extracted, and the slice was physically disrupted and incubated at 4°C overnight, rotating in 500 μL 0.3M NaCl. The liquid was isolated using Spin-X columns, then isopropanol precipitated. Samples were resuspended in 7.5 μL 10 mM Tris-HCl pH 8, then circularized (in 10 μL volume) using CircLigase (Epicentre) according to manufacturer’s instructions. The circularized cDNA was used as a template for PCR using Phusion polymerase and barcoded sequencing primers. Every library had primer NI-NI-2, and each library had one of NI-NI-3, NI-NI-5, NI-NI-6, and NI-NI-7 (primers from [34], and primer sequences are listed in the file S2). PCR was performed 3x on each library—once each for 14, 16, and 18 cycles. Samples were run on an 8% non-denaturing TBE gel at 150V for ~1hr, then the gel was stained with SYBR gold to determine which number of cycles optimized amount of correctly-sized library. For each library, the PCR was repeated in 100 μL volume, and re-run on an 8% non-denaturing TBE gel at 150V (3 lanes for each library). The correct size was gel extracted and the slice was physically disrupted and incubated rotating at 4°C overnight in 500 μL 0.3M NaCl. The samples were isopropanol precipitated and resuspended in 20 μL water.

**Isolation of Natural mRNA Transcripts for Semi-Quantitative Mass Spectroscopy**

50 liters of yeast was grown at 30°C in 2L batches to OD_600_ 0.8-1. Total RNA from the yeast was isolated by the hot-acid phenol method, then ethanol precipitated. RNA from 6-8 liters of yeast was resuspended in 50 mL water. Each 50 mL total RNA was purified twice over columns with 1 g oligo(dT) cellulose (Ambion) as follows: Oligo(dT) cellulose was added to the column, washed 2x with water, and washed 2x with 50 mL binding buffer (0.5M NaCl, 10 mM Tris-HCl pH 7.5, 0.1 mM EDTA). To the 50 mL RNA, 50 mL 2x binding buffer was added, then this mixture was added to the cellulose on the column and rotated at 4°C for 30 min. The column was uncapped and the supernatant was collected, then put back over the column to allow more binding. The column was washed once with 100 mL binding buffer and once with 100 mL wash buffer (0.2 M NaCl, 10 mM Tris-HCl pH 7.5, 1 mM EDTA). Poly(A) RNA was then eluted in 2x 10 mL elution buffer (10 mM Tris-HCl pH 7.5, 1 mM EDTA). This was then ethanol precipitated and resuspended in a minimal volume of water (~250 uL for each 50 mL input RNA). All the poly(A) RNAs were combined (~1.5 mL in all). Specific RNA transcripts were then isolated from this poly(A) RNA as follows: 1.5 mL RNA was heated to 95°C for 5 min, then cooled to 45°C. Meanwhile, 1.5 mL water, 3 mL 2.5x SSPE (375 mM NaCl, 25 mM Na_3_PO_4_, 2.5 mM ETDA) , and 5 nmol each of 2 biotinylated enrichment oligos was heated to 45°C. The RNA was added to the SSPE-oligo premix and heated at 45°C overnight. The oligos were then bound to 300 uL myOne Streptavidin beads (Life Tech) for 1 hr at room temp, then washed once with 5 mL stringent wash buffer (0.1x SSC, 0.1% SDS) at 42°C for 10 min, then once with 5 mL non-stringent wash buffer (0.025x SSC) and twice with 2 mL non-stringent wash buffer for 10 min. The purified mRNAs were then eluted from the beads with 2x 500 mL 85°C water. The RNAs were then precipitated and resuspended in a minimal volume of water. Samples from supernatants, washes, and elutions were taken at every step, and RT-qPCR was used to quantify RPL11a RNA, TEF1/2 RNA, 18S RNA, and 25S RNA at every step to determine level of enrichment and quantity recovered.

**LC-MS/MS on Nucleotides from Natural Yeast mRNAs**

A proper dilution of total RNA corresponding to the amount of background RNA calculated in by qPCR in the natural samples was used in all the control samples. To the +C sample, the proper amount (1-6 pmol, corresponding to how much natural RNA was recovered) of the Ψ spike RNA was added. To the –C sample, the proper amount (1-6 pmol) of IVT RNA (RPL11a or TEF1 RNA) was added. For each of the four samples (+C, -C, Bg, and the natural RNA sample), water was added to 75 uL, ZnCl_2_ was added to a final concentration of 1 mM, and 2uL (2 U) nuclease P1 was added. The samples were incubated at 37°C overnight. To all samples, 9 uL 10x shrimp alkaline phosphatase buffer (Fementas) and 2 uL SAP (Fementas) were added, the samples were incubated at 37°C for 4 hr, and the SAP was inactivated by a 65°C incubation for 15 min. LC-MS/MS was then performed by the Stanford University Mass Spectrometry Facility on these samples to detect U, Ψ, and 2-O-methyl A, G, C, and U.

**Mass Spectroscopy to Check for Change in Coding Potential by the Modified Codon**

The RPL11a-TAP and TEF1-TAP strains were grown to OD_600_ of about 0.8 in 1L of YPD. The yeast were pelleted, washed, and resuspended in buffer B (50 mM Hepes pH 8.0, 140 mM KCl, 1.8 mM MgCl_2_, 0.1% NP-40, 0.5 mM DTT), then lysed by Mini bead-beater 16 from Biospec products (Cat# 607) with four 1-min cycles at max speed. The lysate was cleared by centrifugation for 10 minutes at 8000xg. The concentration of the lysate was diluted to 20 mg/mL, and to each of 4x 1mL of lysate was added 0.5 mL MyONE Streptavidin Dynabeads (Life Tech) coated with IgG biotin. This was incubated at 4°C for 2 hours, then the beads were washed 3x for 15 minutes at 4°C. The first wash contained 4M NaCl, the second 8M urea, and the third 4% sodium lauroyl sarcosinate. The beads were then washed 3x in buffer C (50 mM Hepes pH 8.0, 140 mM KCl, 2 mM EDTA, 0.1% NP-40, 0.5 mM DTT, 10% glycerol), and resuspended in 60 uL Buffer C. Samples were then run on a 4-12% Bis-Tris gel (Bio-Rad). The gel was then stained with GelCode blue (Pierce), and RPL11a-TAP and TEF1-TAP were extracted from the gel. The gel slices were sent to MS Bioworks, where the samples were fragmented with trypsin (RPL11a-TAP) or elastin (TEF1-RAP), and an error-tolerant mass spectrometry was performed to search for any changes in amino acids from the expected amino acid sequence.

**qPCR to Quantify RNA Abundance in Presence or Absence of Pseudouridine Synthases**

3x100 mL each wt, pus1Δ, and pus4Δ strains were grown to log phase as described above. The cells were pelleted, washed in water, and resuspended in TES buffer. RNA was isolated by hot acid phenol chloroform extraction, then isopropanol precipitated and resuspended in 200 μL water (0.9-1.65 μg/μL RNA). Reverse transcription reactions were done using Superscript III with 200 pg RNA in 10 uL. 1 μL of each sample was used for qPCR using Power SYBR Green Master Mix (ABI) on a 7900HT qPCR machine (ABI). The three biological replicates for each experiment were averaged and plotted.

**Quantitative Western Blots to Quantify Protein Abundance in Presence or Absence of Pseudouridine Synthases**

2x250 mL RPL11a-TAP, RPL11a-TAP pus1Δ, TEF1-TAP, TEF1-TAP pus1Δ strains were grown to log phase, heat shock, or stationary phase as explained above (24 total samples). The cells were pelleted, washed in buffer, and resuspended in 500 μL buffer B. Extract was made by bead beating 4x 1 min, then lysates were spun to remove debris. 40 μg total cell extract for each sample was run on a 4-12% Bis-tris gel (Bio-Rad) and transferred to nitrocellulose. Blots were blocked in Odyssey blocking buffer for 30 minutes, then incubated with 1:1500 rabbit anti-CBP (Abcam) and 1:1000 mouse anti-actin (Santa Cruz Biotechnology) or 1:700 mouse anti-tubulin in Odyssey blocking buffer + 0.1% tween. After 3 washes in PBST, samples were incubated in 1:10,000 anti-rabbit IgG 800, 1:1000 anti-mouse IgG 680 in Odyssey blocking buffer + 0.1% tween + 0.01% SDS. After 3 washes in PBST, the blots were dried, and viewed and quantified using an Odyssey scanner.
