# Supplementary figures and images for: Transcriptome-Wide Mapping of Pseudouridines: Pseudouridine Synthases Modify Specific mRNAs in S. cerevisiae
Source: PLoS One. 2014 Oct 29;9(10):e110799. doi: 10.1371/journal.pone.0110799 (PMC4212993; doi:10.1371/journal.pone.0110799)

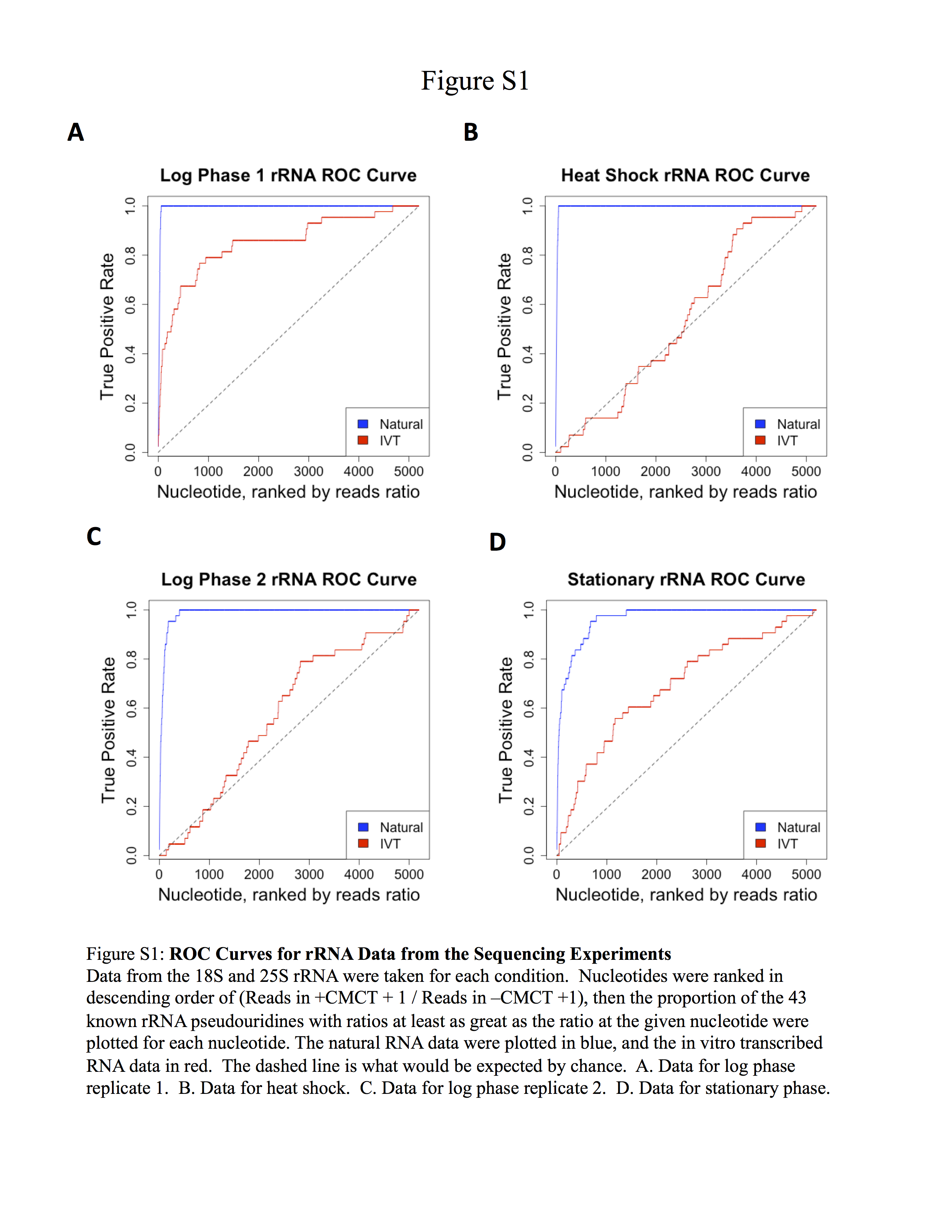

Supplement: File S2 — Supporting figures S1-S9. (TIFF) [file pone.0110799.s004.tiff]
